# Supplementary material for: Comparative Characteristics and Zoonotic Potential of Avian Pathogenic Escherichia coli (APEC) Isolates from Chicken and Duck in South Korea
Source: Microorganisms. 2021 Apr 27;9(5):946. doi: 10.3390/microorganisms9050946 (PMC8145765; doi:10.3390/microorganisms9050946)
Supplement: Supplementary file 1 [file microorganisms-09-00946-s001.zip › microorganisms-1185755-supplementary.pdf]

Table S1. Number of APEC isolates according to sample source.

Host	Liver	Infraorbital sinus	Articular cavity	Air-Sac	Heart	Other sites	Total no. of isolates
Chicken (n=96)	73	9	4	3	3	4	96
Duck (n=29)	29	-	-	-	-	-	29
Total (n=125)	102	9	4	3	3	4	125

Table S2. Primer sets used for detection of virulence-associated genes in the APEC isolates.

Gene function	gene	Description	Primer sequence (5'-3')	Size (bp)	Reference
Adhesins	*fimC*	type 1 fimbriae	F: GGAAATAACATTCTGCTTGC<br>R: TTTGTTGCATCAAGAATACG	288	[17]
	tsh	Temperature-sensitive hemagglutinin	F: GGGAAATGACCTGAATGCTGG<br>R: CCGCTCATCAGTCAGTACCAC	420	[75]
Iron acquisition	*iroN*	Catecholate siderophore (salmochelin) receptor	F: AAGTCAAAGCAGGGGTTGCCCG<br>R: GACGCCGACATTAAGACGCAG	667	[76]
	irp2	Iron-respressible protein (yersiniabactin biosynthetic protein)	F: AAGGATTCGCTGTTACCGGAC<br>R: TCGTCGGGCAGCGTTTCTTCT	280	[77]
	iucD	Iron uptake chelate gene D	F: GTGAGTTGTACCACCGTTTT<br>R: CCATTCCAGAGTGAAGTCAT	278	[17]
	fyuA	Ferric yersiniabactin uptake A	F: CAACATCGTCACCCAGCAG<br>R: CGCAGTAGGCACGATGTTGTA	949	[17]
Toxins	*lt*	Heat-labile toxin	F: ATGAGTACTTCGATAGAGG<br>R: ATG GTATTCCACCTA ACGC	279	[17]
	st	Heat-stable toxin	F: TCTGTATTGTCTTTTTCACCTTTC<br>R: TTAATAGCACCCGGTACAAGC	165	[17]
	stx1A	Shiga toxin	F: CAGTTAATGTGGTGGCGAAG<br>R: CTGCTAATAGTTCTGCGCATC	895	[17]
	stx2A	Shiga toxin	F: CTTCGGTATCCTATTCCCGG<br>R: GGATGCATCTCTGGTCATTG	482	[17]
	Vat	Vacuolating autotransporter toxin	F: TCCTGGGACATAATGGTCAG<br>R: GTGTCAGAACGGAATTGT	981	[78]
	hlyF	Hemolysin F	F: GGCGATTTAGGCATTCCGATACTC<br>R: ACGGGGTCGCTAGTTAAGGAG	599	[76]
Protectins/ Serum resistance	*ompT*	Outer membrane protein T	F: ATCTAGCCGAAGAAGGAGGC<br>R: CCCGGGTCATAGTGTTCATC	559	[76]
	iss	Episomal increased serum survival	F: AGCAACCCGAACCACTTGATG<br>R: TAATAAGCATTGCCAGAGCGG	329	[17]

Table S3. Distribution of O-serogroups of APEC isolates according to sample source and breeds of the chickens and ducks.

Breeds	Liver	Infraorbital sinus	Articular cavity	Air-Sac	Heart	Yolk	Inner ear	Femoral head	Wattle	Total no. of isolates
**Layer**	**17**	**1**		**3**					**1**	**22**
O1	4									4
O2	4									4
O8	1									1
O25	1	1								2
O29	1									1
O45				1						1
O78	1									1
O88	2			1						3
O154									1	1
O166	1									1
NT	2			1						3
**Broiler**	**28**	**7**	**3**		**2**			**1**		**41**
O1	1									1
O2	4									4
O7		1								1
O20					1					1
O21	1									1
O45	2									2
O55					1					1
O68	1									1
O78	2	1								3
O88	1									1
O102	2	1								3
O140			1							1
O177	1	1								2
O182	1		1							2
O184		1								1
NT	12	2	1					1		16
**Broiler breeder**	**12**	**1**	**1**		**1**	**1**	**1**			**17**
O1	1									1
O2	1									1
O8	1									1
O24			1							1
O45	1									1
O51	1									1
O143	1									1
NT	6	1			1	1	1			10
**Korean native chicken**	**16**									**16**
O2	3									3
O5	1									1
O20	1									1
O22	1									1
O24	1									1
O45	2									2
O88	1									1
O115	1									1
NT	5									5
**Chicken**	**73**	**9**	**4**	**3**	**3**	**1**	**1**	**1**	**1**	**96**
**Broiler duck**	**28**									**28**
O1	1									1
O2	1									1
O9	1									1
O15	2									2
O18	1									1
O22	1									1
O24	1									1
O25	2									2
O34	1									1
O39	1									1
O78	3									3
O81	1									1
O89	1									1
O129	1									1
O149	1									1
O150	2									2
O156	1									1
O160	1									1
NT	5									5
**Breeder duck**	**1**									**1**
O84	1									1
**Duck**	**29**									**29**

Table S4. Distribution of multidrug resistant (MDR) APEC isolates from chickens and ducks.

No. of antimicrobial class	Chicken (n=96)		Duck (n=29)		Total	
	No. of isolates	Frequency (%)	No. of isolates	Frequency (%)	No. of isolates	Frequency (%)
8	3	3.1	0	0	3	2.4
7	3	3.1	1	3.4	4	3.2
6	17	17.7	6	20.7	23	18.4
5	27	28.1	3	10.3	30	24.0
4	18	18.8	7	24.1	25	20.0
3	6	6.3	2	6.9	8	6.4
2	11	11.5	3	10.3	14	11.2
1	7	7.3	4	13.8	11	8.8
0	4	4.2	3	10.3	7	5.6
**MDR ≥3**	**74**	**77.1**	**19**	**65.5**	**93**	**74.4**

75. Maurer, J.J.; Brown, T.P.; Steffens, W.; Thayer, S.G. The occurrence of ambient temperature-regulated adhesins, curli, and the temperature-sensitive hemagglutinin tsh among avian *Escherichia coli*. *Avian Dis.* **1998**, *42*, 106–118.

76. Johnson, T.J.; Siek, K.E.; Johnson, S.J.; Nolan, L.K. DNA sequence of a ColV plasmid and prevalence of selected plasmid-encoded virulence genes among avian *Escherichia coli* strains. *J. Bacteriol.* **2006**, *188*, 745–758.

77. Schubert, S.; Rakin, A.; Karch, H.; Carniel, E.; Heesemann, J. Prevalence of the "high-Pathogenicity Island" of *Yersinia* species among *Escherichia* coli Strains that are pathogenic to humans. *Infect. Immun.* **1998**, *66*, 480–485.

78. Ewers, C.; Janßen, T.; Kießling, S.; Philipp, H.-C.; Wieler, L.H. Molecular epidemiology of avian pathogenic *Escherichia coli* (APEC) isolated from colisepticemia in poultry. *Vet. Microbiol.* **2004**, *104*, 91–101.
